# Infant feeding mode predicts the costs of healthcare services in one region of Canada: a data linkage pilot study

**DOI:** 10.1186/s13104-020-05228-6

**Published:** 2020-08-15

**Authors:** Alicia Taylor, Sharmeen Chowdhury, Zhiwei Gao, Hai Van Nguyen, William Midodzi, Nicole Gill, Beth Halfyard, Leigh Anne Allwood Newhook, Laurie Twells

**Affiliations:** 1 Faculty of Medicine, Memorial University of Newfoundland, 300 Prince Philip Drive, St. John’s, NL A1B 3V6 Canada; 2 School of Pharmacy, Memorial University, St. John’s, NL Canada; 3 Newfoundland and Labrador Centre for Health Information, St. John’s, NL Canada; 4 Department of Pediatrics, Faculty of Medicine, Memorial University, St. John’s, NL Canada

**Keywords:** Infant feeding, Breastfeeding, Health Services Research, Healthcare costs, Direct cost, Canada

## Abstract

**Objective:**

The aim is to perform a pilot study evaluating the differences in healthcare service use and its associated costs by infant feeding mode in an infant’s first year of life. Data from a prospective cohort study and administrative databases were linked to examine healthcare use in healthy full term infants (N = 160). Exposure was categorized as exclusively breastfed, mixed fed and exclusively formula fed. Outcomes included hospitalizations, emergency room and physician visits. Descriptive statistics and generalized linear modelling were performed.

**Results:**

Overall $315,235 was spent on healthcare service use for the sample of infants during their first year of life. When compared to exclusive breastfeeding, mixed feeding and exclusive formula feeding were found to be significant predictors of total healthcare service use costs (p < 0.05), driven by costs of hospital admissions. Due to the human and economic burden associated with not breastfeeding, policies and programs that support and encourage breastfeeding should be priority.

## Introduction

Human breastmilk is universally acknowledged as the gold standard of infant feeding, as it offers a unique matrix of compounds for optimal growth, health and development in a newborn child [1]. Studies show that breastfeeding reduces the rates of infant morbidity and mortality, as well as decreases the risk of chronic illnesses in childhood [2–6]. The World Health Organization (WHO) and Health Canada recommend mothers exclusively breastfeed their infants to 6 months of age, with continued breastfeeding with complementary foods to 2 years of life and beyond [7, 8]. Research in developed countries has also examined the economic benefit of increasing breastfeeding rates [9–12]. Researchers demonstrated how increasing breastfeeding rates to 90% of exclusive breastfeeding (EBF) at 6 months has the potential to prevent $3 billion in direct medical costs, and $14.2 billion premature death costs, annually [9]. In the United Kingdom, researchers estimated that £26.8 million could be gained annually by avoiding the costs of treating acute infections in infancy and breast cancer in mothers, if EBF rates increased to 65% at 4 months and 100% of babies were breastfed at hospital discharge [10]. Similarly, Australian hospital systems estimated costs of $1–2 million annually for the treatment of common childhood infections due to insufficient breastfeeding [11]. In Canada, little is known about the economic impact of increasing breastfeeding rates, as few studies have examined the impact of infant feeding mode (IFM) on healthcare service use (HSU) [13–16]. Two studies concluded that breastfeeding was strongly protective against infections severe enough to require hospital admission; however, both studies were small and focused on Indigenous populations [13–15], and neither study conducted a cost analysis related to HSU. Therefore we sought to conduct a pilot study examining the impact of IFM on HSU costs during an infant’s first year of life (YOL) in one region of Canada.

## Main text

### Materials and methods

#### Sample

Methods from the F*eeding infants in Newfoundland and Labrador* (FiNaL) study have been previously published [17–19]. In brief, the study was conducted in three phases to evaluate maternal attitudes and infant feeding practices in Newfoundland and Labrador (NL), Canada. Of those who completed phase three of the FiNaL study (n = 554), 362 healthy full term infants born in the Eastern Health Region of NL were eligible for the current pilot evaluating HSU. Ineligibility included preterm infants, those unable to breastfeed due to illness (i.e. congenital disorders), multiple births, deceased, or parental refusal. Of this sample, 160 mothers provided their infants health insurance number for the data linkage.

### Administrative data

The series of administrative data was created by the NL Centre for Health Information (NLCHI). Individual level record data were extracted from the Provincial Discharge Abstract Database (PDAD) for hospital admissions, Live Birth System (LBS) for demographics and infant characteristics, MCP Fee-for-Service Physician Claims (FFS) for family doctor and specialist visits, and the MediTech Module (MTER) for emergency room (ER) visits.

### Exposure

IFM was defined using the WHO criteria. ‘Exclusive breastfeeding (EBF)’ defined infants receiving only breast milk (i.e., expressed or from a wet nurse) and nothing else, except for oral rehydration solution, medicines, vitamins and minerals when needed. ‘Mixed feeding’ (MF) defined infants receiving breastmilk and other food or liquid including water, non-human milk, and formula. ‘Exclusive formula feeding’ (EFF) defined infants fed only breastmilk substitutes. Due to the self-reporting of breastfeeding, EBF could only be valid for the first month of life, though information on IFM was collected throughout the first YOL.

### Outcomes

Primary outcomes were HSU and the associated costs for hospital admissions, ER and physician visits. Direct HSU costs reflect those to the payer over a 1 year time horizon.

### Statistical analysis

Deterministic linkage was used to link the FiNaL study and administrative data provided by the NLCHI. Descriptive statistics, either frequencies for categorical variables or means and standard deviations (SD) for continuous variables were conducted. Generalized linear modelling (GLM) was used to compare HSU costs, and maternal and child characteristics between IFM. Billable claims for each healthcare provider visit were summed using the claims provided by the NLCHI. Costs were converted to 2015 Canadian dollars, as all resource intensity weights (RIW) and cost per standard hospital stay (CSHS) used were for the 2015 fiscal year. The average cost of an ER visit for the EH regional health authority was calculated using the average of the 2014/2015 and 2015/2016 fiscal years. Due to the skewed HSU costs, both base case and robustness GLM analyses were performed. For the base case GLM a gamma distribution and a log link function were used. Following findings from the modified park and box cox tests, the robustness check followed an inverse gaussian distribution and a reciprocal function. A power calculation was conducted for the GLM at an alpha level of 0.05, by IFM; EBF (n = 107), MF (n = 32), EFF (n = 21). Statistical analysis was conducted using SPSS (IBM 25) software [20].

## Results

There were 160 infant health insurance numbers provided for the data linkage. The recruitment process is illustrated in the Additional file 1. The majority of mothers were Caucasian, 26 years of age or older, partnered, with a household income > $30,000 CAN and a post-secondary education. Maternal characteristics are outlined in Table 1. Consenting mothers were more likely to be older, partnered, with higher levels of education and household income. All infants were full term (i.e., > 37 weeks gestation), born between 2012 and 2014, with a mean birth weight of 3523.5 g (SD 455.8). IFM was categorized as 67% EBF to 1 month, 20% MF, and 13% EFF. There were no differences when examining infants gender, and appropriateness of size for gestational age between groups of IFM, p > 0.05.

**Table 1:** Maternal characteristics (frequency, n (%))

Characteristics	PN1 survey (N = 554)	No MCP (N = 394)	HSU [admin] (N = 160)	P value*
Infant feeding mode				
EBF at 1 month	291 (52.5%)	201 (51.0%)	107 (66.9%)	*0.003*
Mixed fed	165 (29.8%)	116 (29.4%)	32 (20.0%)	
EFF	98 (17.7%)	77 (19.5%)	21 (13.1%)	
Mother’s age (> 26 years)	494 (89.2%)	344 (86.8%)	152 (95.0%)	*0.005*
Marital status (married/partnered)	518 (93.7%)	362 (92.1%)	156 (97.5%)	*0.018*
Education level (post-secondary)	510 (92.1%)	354 (89.8%)	156 (97.5%)	*0.003*
Household income (> 30,000 CAN$)	520 (93.9%)	363 (92.1%)	157 (98.1%)	*0.008*
Parity (primiparous)	316 (57.2%)	218 (55.6%)	98 (61.3%)	0.224
Type of delivery (vaginal)	403 (73.0%)	286 (73.0%)	117 (73.1%)	0.968
Smoking status (current)	18 (3.20%)	16 (4.1%)	2 (1.30%)	0.091
Dwelling area (urban)	253 (45.7%)	136 (44.9%)	117 (73.1%)	*0.0000*
Ethnicity: caucasian	514 (94.5%)	362 (94.0%)	151 (95.6%)	0.478
Ethnicity: other (i.e., Asian, Aboriginal, Afro-Canadian)	30 (5.5%)	23 (5.9%)	7 (4.4%)	

*P value compares those that took part in the HSU study (n = 160) to those that did not (n = 394)

### Healthcare service use

The majority of infants were seen by a family doctor (n = 151) within their first YOL, irrespective of feeding mode, and over half (n = 92) were seen by a specialist. MF and EFF infants had a higher percentage of specialist visits (65.6% and 65.0%, respectively), while 55.8% of EBF infants visited a specialist during the first year. The highest frequency of billable claims was: no specific illness diagnosed at the visit (n = 591), signs and symptoms not otherwise diagnosed as an infection or disease (n = 225), upper respiratory tract infection (n = 92), otitis media (n = 61), common cold (n = 49), disorders of eyes and ears (n = 46), thrush (n = 46), and atopic dermatitis (n = 37). There were 12 infants hospitalized at least once during the first YOL. The length of stay (LOS) ranged from 1 to 7 days. Most commonly, admissions were related to respiratory complications (i.e., upper respiratory tract infection, croup, asthma). EBF infants had fewer hospital admissions (2.8%) than that of MF (15.2%) and EFF (19.0%), p < 0.05. Half of infants (n = 83) were brought to the ER at least once during the firstYOL. More MF infants had ER visits (56.3%) than that of EBF (53.3%) and EFF infants (38.1%). MF and EBF infants had significantly more ER visits when compared to EFF infants, p < 0.01. Additional information regarding hospital admissions, ER and physician visits can be found in the Additional file 1.

### Cost associated

The direct HSU costs of 160 healthy full term infants during their first YOL amounted to $315,235.56 (Table 2). When considering costs associated with HSU post discharge from birth, the expenditures equated to $127,373.41. The highest percentage spent on HSU was for hospital admissions, 37.6%, ($47,867.56), followed by visits to the family doctor, 30.1%, ($38,271.88), emergency room 18.8% ($23,805.5) and specialists, 13.7% ($17,254.3) There were no differences between IFM when comparing physician services or ER visits, p > 0.05, while EFF infants had higher expenses for hospital admissions than other feeding groups (MF, EBF), p = 0.010 (Additional file 1).

**Table 2:** Total costs associated with each healthcare provider, by infant feeding mode

	Total (n = 156)	EBF (n = 104)	Mixed (n = 32)	Formula (n = 20)	P Value
Hospitalizations (n = 159)	$47,867.56	$5132.90	$24,823.42	$17,911.24	0.141
Hospitalizations (n = 159) (including birth)	$235,883.92	$128,434.74	$64,126.26	$43,322.92	*0.015*
Emergency room (n = 160)	$23,805.50	$16,264.60	$4731.52	$2809.34	0.672
Family doctor and specialist (n = 156)	$55,546.18	$36,465.19	$10,949.68	$8131.31	0.972
Total costs	$127,373.41	$58,345.41	$40,255.41	$28,851.89	
Total costs (including birth):	$315,235.56	$181,164.53	$79,807.46	$54,263.57	

*P value compares all three groups of infant feeding mode

### Multivariate analysis

Our sample size provides a power level of 78% (alpha 0.05) to examine the primary outcome of total HSU costs. The base case analysis for the GLM is presented in Table 3. IFM remained a predictor of total HSU costs during an infant’s first YOL, after adjustment for residence (urban/rural), delivery type (vaginal/caesarean section), and parity (primiparous/multiparous). Compared to EBF (to 1 month), both MF and EFF were significant predictors of higher total HSU costs. No other factors were significantly associated with total HSU costs. Based on our robustness check the statistical significance remained, with IFM predictive of total HSU cost.

**Table 3:** Generalized linear modelling of total healthcare costs during the infant’s first year of life

	Base case analysis		
	Coefficient (SE)	95% CI	P value
Constant	7.408 (0.889)	7.234–7.582	0.000
Infant feeding mode			
EFF	0.383 (0.118)	0.152–0.615	0.001
MF	0.408 (0.099)	0.212–0.603	0.000
EBF	(Referent)		
Residence			
Rural area	0.124 (0.090)	− 0.52–0.301	0.167
Urban area	(Referent)		
Parity			
Multiparous	0.061 (0.080)		
Primiparous	(Referent)	− 0.096–0.218	0.444
Delivery type			
Vaginal	− 0.042 (0.088)	− 0.214–0.130	0.629
Caesarean section	(Referent)		

*EFF, exclusive formula feeding; MF, mixed feeding; EBF, exclusive breastfeeding

## Discussion

In the present study, 160 mother-infant dyads were enrolled in a data linkage pilot examining the impact of IFM on total HSU costs. Cumulative HSU cost in the first YOL for all healthy full term infants in one provincial region in Canada was $315, 235.56, including cost of birth. The highest percentage spent on HSU was for hospital admissions, followed by family doctor, ER and specialist visits. Higher HSU costs were associated with EFF infants when examining hospitalizations (birth and admissions), and significant differences were found between IFM when examining total HSU costs.

Our findings are consistent with the literature, where studies in other countries have shown that infants who had early exposure to formula experienced higher volumes of visits to family doctors, infectious episodes and hospital admissions [21–25]. Similarly, our study found differences when comparing IFM, where EFF infants had higher average spending associated with hospital admissions, family doctor and specialist visits, and both MF and EFF infants were predictive of higher total HSU costs. The WHO recommends EBF for 6 months for full health benefits, and our study demonstrates that differences can be seen between IFM even when EBF to 1 month.

There are a number of strengths of the current study. To our knowledge, this is the first estimate of HSU costs by IFM of a sample of full term healthy infants living in Canada. Our ability to link maternal and child data allowed us to examine the specific characteristics that are associated with higher HSU costs among infant’s (i.e., mother’s parity, type of delivery and residence), and control for these covariates in our analysis. The advantage of conducting a pilot study has allowed us to assess the feasibility of collecting data in a Canadian context and estimate variability in outcomes to help determine sample size for future larger provincial studies. The administrative database allowed for the calculation of individual level data and the direct costs associated with HSU through the claims of family doctors and specialists.

## Conclusions

In one region of Canada, EFF and MF were found to be significant predictors of the total HSU costs during the first YOL. Further research is needed in a Canadian context examining larger samples, with a necessity for more reliable and valid measures of exposure to capture longer durations of IFM. Due to the human and economic burden associated with no breastfeeding, policies and programs that support and encourage breastfeeding should be a priority for governments and regional health authorities.

## Limitations

Our results are based on a relatively small sample size, where the subsample of the FiNaL Study had a selection bias of primiparous, Caucasian, higher education and household income mothers. We found differences among groups for the total HSU costs, driven mainly by hospitalizations, however significant differences were not observed when examining the costs associated with other HSU (i.e., ER, Physician visits). This could be explained by either having no differences among groups, or that the study was underpowered to examine the differences of ER and physician visits. Due to challenges with collecting exposure data on feeding mode and its duration, our MF and EFF covered the first 6 months of life, but our EBF rate was considered valid and reliable for the first month only. The data on exposure were self-reported by mothers and therefore could result in misclassification. Based on the health insurance claims in NL, the administrative databases can only collect information on FFS physicians. Therefore there are a proportion of family doctors and pediatricians that are salaried that we would not have HSU on, therefore this an underestimate of the use and cost experienced in this region. In addition, although we used a health systems perspective to examine the costs, not all costs were included (i.e., medications).

## Supplementary information

**Additional file 1: Figure S1.** Flowchart of participant requirement process**. Table S1.** Healthcare Provider Visits by Infant Feeding Mode (Frequency, n (%)). **Table S2.** Number of Unique Visits to Healthcare Providers and the Associated Costs by IFM (Counts). **Table S3.** Description of Hospitalizations During The First Year of Life. **Table S4.** Mean (SD), Median (IQR), Min & Max of the Total Costs Associated with each Healthcare Provider, By Infant Feeding Mode.

## Data Availability

The administrative data that support the findings of this study are available from the Newfoundland and Labrador Centre for Health Information (NLCHI) but restrictions apply to the availability of these data, which were used under license for the current study, and so are not publicly available. Data are however available from the authors upon reasonable request and with permission of the NLCHI. The prospective cohort (FiNaL Study) datasets used and/or analysed during the current study are available from the corresponding author on reasonable request.

## Abbreviations

BFRWG: Breastfeeding Research Working Group
CMG: Case Mix Group
CSHS: Cost of a standard hospital stay
EBF: Exclusively breastfeeding
EFF: Exclusively formula feeding
ER: Emergency room
FiNaL Study: Feeding infants in Newfoundland and Labrador
GLM: Generalized Linear Modelling
HCP: Healthcare provider
HSU: Healthcare service use
IFM: Infant feeding mode
IQR: Interquartile range
KW: Kruskal-Wallis
LBS: Live birth system
PDAD: Provincial Discharge Abstract Database
FFS: Medical Care Plan Fee-for-Service Physician Claims
MCP: Medical care plan
MF: Mixed feeding
MTER: MediTech Emergency Room
NL: Newfoundland and Labrador
NLCHI: Newfoundland and Labrador Centre for Health Information
NL SUPPORT: Newfoundland and Labrador support for people and patient oriented research trials unit
TPMI: Translational personalised medicine initiative
RIW: Resource intensity weight
SD: Standard deviation
WHO: World Health Organization
YOL: Year of life
