# Supplementary material for: Infant feeding mode predicts the costs of healthcare services in one region of Canada: a data linkage pilot study
Source: BMC Res Notes. 2020 Aug 15;13:385. doi: 10.1186/s13104-020-05228-6 (PMC7429700; doi:10.1186/s13104-020-05228-6)
Supplement: Supplementary file 1 — Additional file 1: Figure S1. Flowchart of participant requirement process. Table S1. Healthcare Provider Visits by Infant Feeding Mode (Frequency, n (%)). Table S2. Number of Unique Visits to Healthcare Providers and the Associated Costs by IFM (Counts). Table S3. Description of Hospitalizations During The First Year of Life. Table S4. Mean (SD), Median (IQR), Min & Max of the Total Costs Associated with each Healthcare Provider, By Infant Feeding Mode. [file 13104_2020_5228_MOESM1_ESM.docx]

**Additional file 1**

**
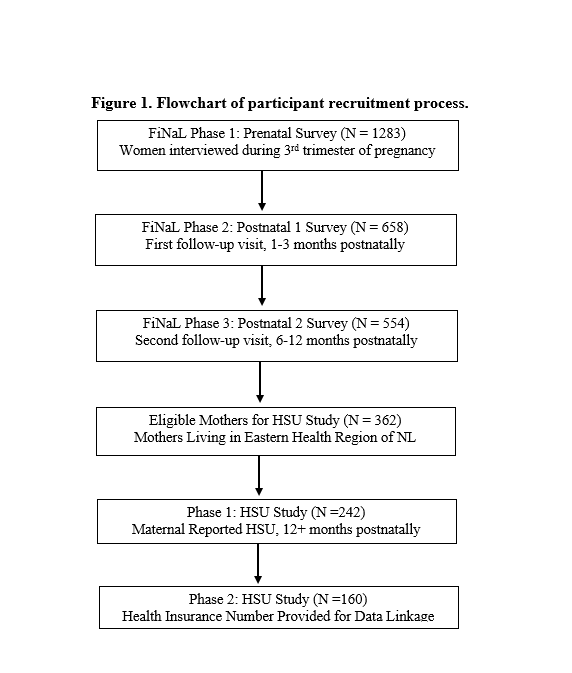
**

| **Table S1. Healthcare Provider Visits by Infant Feeding Mode (Frequency, n (%))** | | | | |
| --- | --- | --- | --- | --- |
|  | **Total (n=159)** | **EBF (n = 106)** | **Mixed (n= 32)** | **Formula (n=21)** |
| **Hospitalization (n=159)** | 12 (7.5) | 3 (2.8) | 5 (15.2) | 4 (19.0) |
|  | **Total (n=160)** | **EBF (n = 107)** | **Mixed (n= 32)** | **Formula (n=21)** |
| **Emergency Room (n=160)** | 83 (51.9) | 57 (53.3) | 18 (56.3) | 8 (38.1) |
|  | **Total (n = 156)** | **EBF (n = 104)** | **Mixed (n= 32)** | **Formula (n=20)** |
| **Family Doctor (n=156)** | 151 (96.8) | 100 (96.2) | 32 (100.0) | 19 (95.0) |
| **Any Specialist (n=156)** | 92 (59.0) | 58 (55.8) | 21 (65.6) | 13 (65.0) |
| **Pediatrician** | 21 (13.5) | 15 (14.4) | 4 (12.5) | 2 (10.0) |
| **Pediatric Cardiologist** | 8 (5.1) | 5 (4.8) | 2 (6.3) | 1 (5.0) |
| **Dermatologist** | 11 (7.1) | 4 (3.8) | 3 (9.4) | 4 (20.0) |
| **Diagnostic Radiologist** | 68 (43.6) | 42 (40.4) | 17 (53.1) | 9 (45.0) |
| **Plastic Surgeon** | 2 (1.3) | 0 (0.0) | 1 (3.1) | 1 (5.0) |
| **Otolaryngologist (ENT)** | 9 (5.8) | 6 (5.8) | 2 (6.3) | 1 (5.0) |

| **Table S2. Number of Unique Visits to Healthcare Providers and the Associated Costs by IFM (Counts)** | | | | |
| --- | --- | --- | --- | --- |
|  | **Total (n=159)** | **EBF (n = 106)** | **Mixed (n= 32)** | **Formula (n=21)** |
| **Hospitalizations** | 15 | 3 | 6 | 6 |
| **Total L.O.S (days)**  **Mean (SD)** | 31  2.58 (2.43) | 6  2.0 (1.0) | 9  1.8 (0.84) | 16  4.0 (2.16) |
| **Total L.O.S (days)**  **including birth**  **Mean (SD)** | 386  2.42 (1.28) | 227  2.14 (0.97) | 95  2.96 (1.18) | 64  3.04 (2.11) |
|  | **Total (n=160)** | **EBF (n = 107)** | **Mixed (n= 32)** | **Formula (n=21)** |
| **Emergency Room Visit** | 161 | 110 | 32 | 19 |
| **Triage Level of ER Visits** |  |  |  |  |
| Emergent (Within 15min) | 6 | 4 | 2 | 0 |
| Urgent (Within 30min) | 58 | 39 | 14 | 5 |
| Less Urgent (Within 60 min) | 59 | 46 | 8 | 5 |
| Non Urgent (in 120 min) | 13 | 6 | 3 | 4 |
| No Triage Level Listed | 25 | 15 | 5 | 5 |
|  | **Total (n=156)** | **EBF (n = 104)** | **Mixed (n= 32)** | **Formula (n=20)** |
| **Family Doctor** | 1101 | 711 | 237 | 153 |
| **Any Specialist** | 220 | 130 | 54 | 36 |
| **Pediatrician** | 46 | 30 | 7 | 9 |
| **Pediatric Cardiologist** | 15 | 10 | 4 | 1 |
| **Dermatologist** | 18 | 4 | 7 | 7 |
| **Diagnostic Radiologist** | 117 | 74 | 30 | 13 |
| **Plastic Surgeon** | 4 | 0 | 1 | 1 |
| **Otolaryngologist (ENT)** | 20 | 12 | 5 | 3 |

| **Table S3. Description of Hospitalizations During The First Year of Life** | | | | | |
| --- | --- | --- | --- | --- | --- |
| **Hospitalizations** | **Admission** | **LOS** | **Case Mix Group (CMG)** | **Description** | **Admit Category** |
| 997 (Mixed) | 1^st^ | 4 | Major Respiratory Complication | Bronchiolitis Resp Syncytial Virus | Urgent/Emergent |
|  | 2^nd^ | 1 | Hernia Repair | Undescended Testicle | Elective |
| 65 (EFF) | 1^st^ | 5 | Upper/Lower RTI | Bronchiolitis Resp Syncytial Virus | Urgent/Emergent |
|  | 2^nd^ | 3 | Upper/Lower RTI | Bronchiolitis unspecified | Urgent/Emergent |
| 1285 (EFF) | 1^st^ | 4 | Fever | Fever | Urgent/Emergent |
| 178 (Mixed) | 1^st^ | 1 | Fever | Fever | Urgent/Emergent |
| 199(EFF) | 1^st^ | 1 | Other Resp Problem | Dyspnoea | Urgent/Emergent |
|  | 2^nd^ | 1 | Other Resp Problem | Dyspnoea | Urgent/Emergent |
| 445 (EFF) | 1^st^ | 2 | Asthma | Asthma unspecified | Urgent/Emergent |
| 901 (Mixed) | 1^st^ | 2 | Gastrointestinal Hemorrhage | Haematemesis | Urgent/Emergent |
| 128 (EBF) | 1^st^ | 1 | Croup | Croup - Acute Obstructive laryngitis | Urgent/Emergent |
| 372 (Mixed) | 1^st^ | 1 | Hand Intervention | Upper Limb  (Accessory Finger) | Elective |
| 883 (EBF) | 1^st^ | 2 | Jaundice | Jaundice Unspecified | Urgent/Emergent |
| 887 (Mixed) | 1^st^ | 2 | Short Gestation | Unspecified | Newborn |
| 1214 (EBF) | 1^st^ | 2 | Jaundice | Jaundice Unspecified | Urgent/Emergent |

| **Table S4. Mean (SD), Median (IQR), Min & Max of the Total Costs Associated with each Healthcare Provider, By Infant Feeding Mode** | | | | | |
| --- | --- | --- | --- | --- | --- |
| **Hospitalizations** | **Total (n = 156)** | **EBF (n = 104)** | **Mixed (n= 32)** | **Formula (n=20)** | **P Value** |
| MEAN (SD) | 3988.96 (3188.99) | 1710.97 (122.67) | 4964.68 (4415.57) | 4477.81 (2046.37) | 0.141 |
| MEDIAN (IQR) | 3040.90 (3778.43) | 1781.79 (213.48) | 3805.69 (6037.35) | 4580.27 (3728.46) |  |
| MIN - MAX | 1430.60 - 12,664.22 | 1569.31 - 1781.79 | 1430.60 - 12,664.22 | 2402.84 - 6347.87 |  |
| **Hospitalizations**  **(including birth)** | **Total (n = 156)** | **EBF (n = 104)** | **Mixed (n= 32)** | **Formula (n=20)** |  |
| MEAN (SD) | 1483.55 (1434.19) | 1211.65 (492.40) | 2003.95 (2519.00) | 2062.99 (1990.91) | **0.015** |
| MEDIAN (IQR) | 1202.99 (530.25) | 900.35 (481.70) | 1382.06 (706.16) | 1382.06 (851.18) |  |
| MIN - MAX | 900.35 - 14,329.37 | 900.35 - 3791.20 | 900.35 - 14,329.37 | 900.35 - 7501.69 |  |
| **Emergency Room** | **Total (n = 156)** | **EBF (n = 104)** | **Mixed (n= 32)** | **Formula (n=20)** |  |
| MEAN (SD) | 148.78 (220.31) | 152.00 (214.42) | 147.86 (246.27) | 133.77 (218.83) | 0.672 |
| MEDIAN (IQR) | 147.86 (147.86) | 147.86 (295.72) | 147.86 (147.86) | 147.86 (221.79) |  |
| MIN - MAX | 147.86 -1478.6 | 147.86 -1478.6 | 147.86 -1330.74 | 147.86 - 739.30 |  |
| **Family Doctor & Specialist** | **Total (n = 156)** | **EBF (n = 104)** | **Mixed (n= 32)** | **Formula (n=20)** |  |
| MEAN (SD) | 356.07 (288.42) | 350.63 (266.23) | 342.18 (254.37) | 406.57 (430.91) | 0.972 |
| MEDIAN (IQR) | 300.12 (307.85) | 296.73 (339.74) | 291.40 (307.30) | 301.92 (194.61) |  |
| MIN - MAX | 6.40 - 2065.68 | 6.40 - 1678.90 | 25.63 - 1244.26 | 54.14 - 2065.68 |  |
| *P Value compares all three groups of Infant Feeding Mode | | | | | |
